# The Cornelia de Lange Syndrome-associated factor NIPBL interacts with BRD4 ET domain for transcription control of a common set of genes

**DOI:** 10.1038/s41419-019-1792-x

**Published:** 2019-07-18

**Authors:** Noelia Luna-Peláez, Rosana March-Díaz, María Ceballos-Chávez, Jose A. Guerrero-Martínez, Paolo Grazioli, Pablo García-Gutiérrez, Thomas Vaccari, Valentina Massa, Jose C. Reyes, Mario García-Domínguez

**Affiliations:** 1 Andalusian Center for Molecular Biology and Regenerative Medicine-CABIMER, CSIC-Universidad de Sevilla-Universidad Pablo de Olavide, Av. Américo Vespucio 24, 41092 Seville, Spain; 2 Dipartimento di Scienze della Salute, Università degli Studi di Milano, Via A. di Rudinì 8, 20142 Milano, Italy; 3 Dipartimento di Bioscienze, Università degli Studi di Milano, Via Giovanni Celoria 26, 20133 Milano, Italy

**Keywords:** Transcriptional regulatory elements, Experimental models of disease

## Abstract

Mutations in *NIPBL* are the major cause of Cornelia de Lange Syndrome (CdLS). NIPBL is the cohesin-loading factor and has recently been associated with the BET (bromodomains and extra-terminal (ET) domain) proteins BRD2 and BRD4. Related to this, a CdLS-like phenotype has been described associated to *BRD4* mutations. Here, we show direct interaction of NIPBL with different BET members in yeast, and selective interaction with BRD4 in cells, being the ET domain involved in the interaction. To understand the relationship between NIPBL and BET proteins, we have performed RNA-Seq expression analysis following depletion of the different proteins. Results indicate that genes regulated by NIPBL largely overlap with those regulated by BRD4 but not with those regulated by BRD2. ChIP-Seq analysis indicates preferential NIPBL occupancy at promoters, and knockdown experiments show mutual stabilization of NIPBL and BRD4 on co-regulated promoters. Moreover, human fibroblasts from CdLS probands with mutations in *NIPBL* show reduced BRD4 at co-occupied promoters. Functional analysis in vivo, using mutants of *Drosophila melanogaster*, confirmed the genetic interaction between *Nipped-B* and *fs(1)h*, the orthologs of human *NIPBL* and *BRD4*, respectively. Thus, we provide evidence for NIPBL and BRD4 cooperation in transcriptional regulation, which should contribute to explain the recently observed CdLS-like phenotype associated with *BRD4* mutations.

## Introduction

Bromodomain and extra-terminal domain (BET) proteins are chromatin readers with an important role in cell cycle progression^1–3^. The BET family in vertebrates comprises BRD2, BRD3, BRD4, and BRDt. With the exception of the testis-specific member BRDt, BET proteins are widely expressed during development and in the adult. The observation that some BET members remain associated to chromosomes during mitosis has led to hypothesize that BET proteins act as true epigenetic factors marking key genes across generations^4,5^. Two tandem bromodomains at the N-terminus are involved in recognition of acetyl groups in proteins, notably histones^6–9^. The prominent role that these proteins display in cell cycle control has boosted the development of drugs antagonizing BET proteins, as an effective therapy against a variety of cancer types^10,11^. Thus, synthetic molecules mimicking acetyllysine groups, which are able to efficiently dissociate BET proteins from the chromatin, have been successfully used for tumors treatment in mice^12–17^. Besides the bromodomains, a conserved motif B (mB) accounts for protein dimerization^18^, while the conserved and exclusive extra-terminal (ET) domain involved in interaction with other proteins^19^ is distinctive of these proteins and defines them as a family. In contrast to BRD2 and BRD3, BRD4 presents an additional C-terminal domain (CTD) that is essential for its function^20^.

A preserved structure among the different BET proteins and an elevated homology at the level of functional domains, together with overlapping expression patterns due to ubiquitous expression, have risen the question about functional redundancy between members. However, *Brd2* and *Brd4*, the most studied BET genes, appear essential and nonredundant in vertebrates, considering that single knockout mice were found to be embryonic lethal^21–23^. In addition, while BRD4 exerts an important role in transcription elongation as a component of the transcription elongation complex P-TEFb^24^, BRD2 has been mostly involved in transcription initiation^2,25,26^. Finally, both proteins have been associated with the Mediator complex and with different chromatin remodeling machineries^19,27–29^.

On the basis of different genomic occupancy, BRD2, but not BRD4, was recently reported to associate with CTCF and the cohesin complex to support cis-regulatory enhancer assembly during transcription activation^30^. Among cohesin complex proteins co-precipitating with BRD2 is NIPBL, the cohesin-loading factor^30^. Heterozygous mutations in *NIPBL* account for about 60% of the cases of Cornelia de Lange syndrome (CdLS), a genetic disorder with multiple abnormalities including growth and mental retardation, inner organ malformations and a typical *facies*^31^. Mutation of *BRD4* was also recently described to cause a CdLS-like phenotype, establishing a link between NIPBL and BRD4^32^. However, the molecular mechanism behind these interactions remains unexplored. Therefore, at present, although a role in transcription has been indicated for NIPBL^33–36^, the relationship between BRD2 or BRD4 and CdLS is unclear.

From a two-hybrid screening previously performed^18^ we identified NIPBL as a BRD2 partner. Here, we report NIPBL interaction with different BET members in yeast. However, immunoprecipitation (IP) experiments indicate preferential association with BRD4 in mammal cells. To establish functional association of NIPBL with BRD2 or BRD4, we have compared cell transcriptomes following knockdown of *Nipbl*, *Brd2*, or *Brd4*. Results reveal a marked overlap in downregulated genes between cells depleted of NIPBL and BRD4. Chromatin immunoprecipitation (ChIP) experiments in cell lines and primary fibroblasts from CdLS patients with mutations in *NIPBL* indicate mutual stabilization of both proteins at co-regulated promoters, and analysis in *Drosophila* indicate genetic interaction between NIPBL and BET coding genes. Thus, our data strongly support a functional cooperation between NIPBL and BRD4 in regulating gene expression at the promoter level.

## Results

### NIPBL interacts with the ET domain of BET proteins

**Fig. 1:**
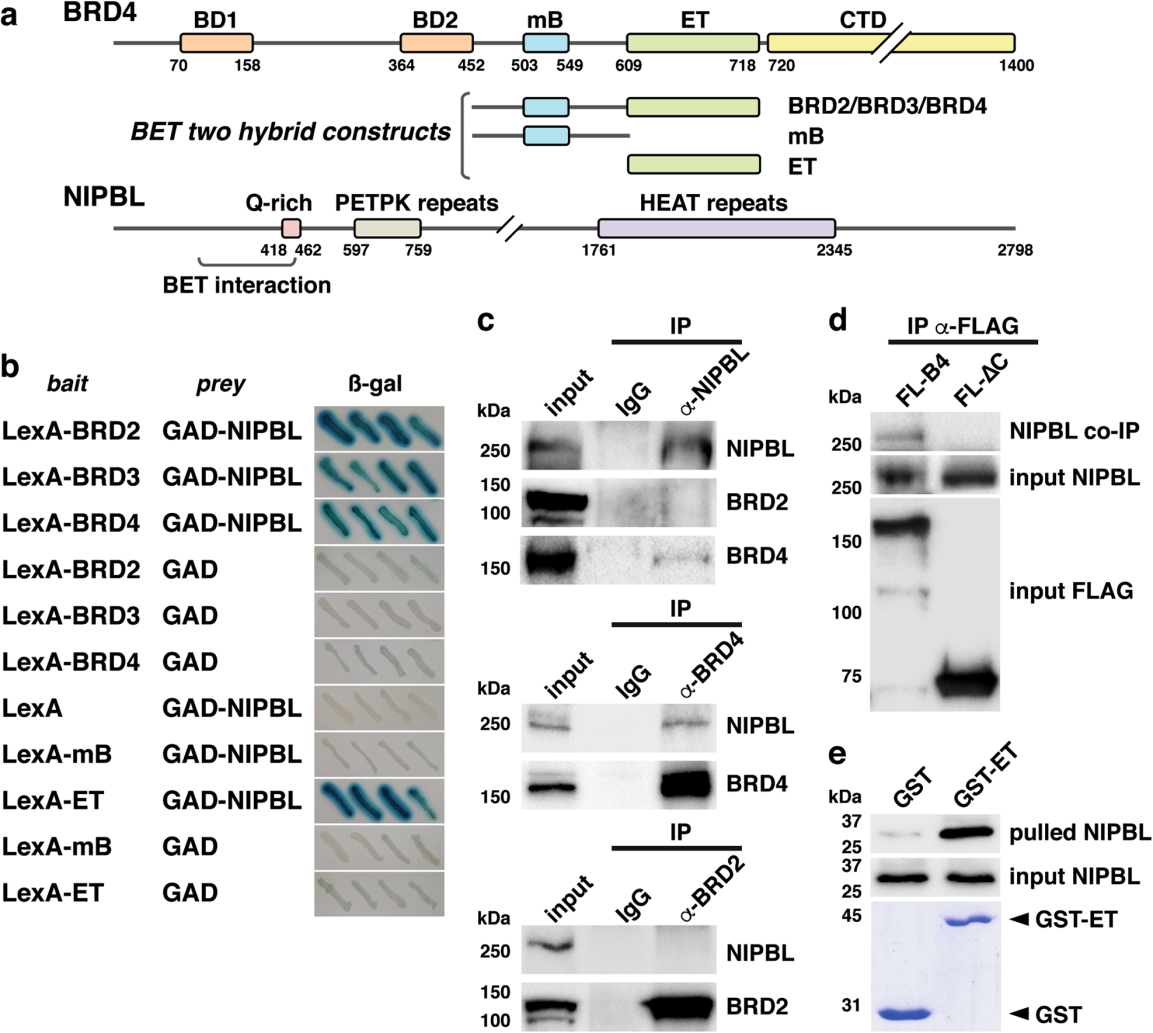
NIPBL interacts with BRD4 through the ET domain. **a** Schematic representation of the BET two-hybrid constructs used and the region of NIPBL, as identified in the prey clone (amino acids 212–449), interacting with BET proteins. Numbers correspond to amino acid positions in the mouse sequence. A graphic map of BRD4 with main domains is shown as a BET example. Relevant domains of NIPBL are also shown. BD1 and BD2 bromodomains 1 and 2, mB motif B, ET extra-terminal domain, CTD C-terminal domain, Q-rich glutamine rich domain. **b** The ß-galactosidase (ß-gal) assay on yeast harboring the indicated prey and bait constructs. Blue color, interaction; white color, no interaction. **c** The immunoprecipitation assay of endogenous NIPBL, BRD4, and BRD2 proteins analyzed by western blot after immunoprecipitation (IP) with NIPBL, BRD4, or BRD2 antibodies (α-) or whole rabbit IgG. Input corresponds to 5% of the precipitated cell extract. **d** The immunoprecipitation assay to analyze by western blot co-immunoprecipitated (co-IP) NIPBL with FLAG antibodies (α-FLAG) after transfection of FLAG-BRD4 (FL-B4) and FLAG-BRD4∆C (FL-∆C) expression constructs. Inputs correspond to 5% of the precipitated cell extract. **e** The pull-down assay of *E. coli*-purified GST or a GST-ET fusion incubated with a FLAG-tagged N-terminal NIPBL peptide. Retained and input (20%) NIPBL were revealed by western blot with FLAG antibodies, while input GST and GST-ET (100%) were revealed by Coomassie blue staining

Searching for BRD2 partners, we previously performed a two-hybrid screening by testing a truncated BRD2 bait construct, which lacks the bromodomains, against an 11-day-old mouse embryo cDNA library^18^. We identified up to eight different proteins interacting with BRD2, being the cohesin-loading factor NIPBL one of them. The NIPBL interacting fragment that we isolated mapped to the N-terminus (Fig. 1a). Two-hybrid indicated that the interaction was not restricted to BRD2, since similar constructs based on BRD3 and BRD4, also interacted with NIPBL (Fig. 1b). Two-hybrid results also indicated that BRD4 CTD was dispensable for NIPBL interaction (Fig. 1b). Notably, mapping of the BET domain, which mediates the interaction, revealed the involvement of the ET domain rather than the region containing the mB (Fig. 1b).

We next conducted IP experiments to analyze the newfound interaction in the context of endogenous proteins. For this purpose we precipitated NIPBL from HEK293T cells extracts and analyzed co-precipitation of BRD2 and BRD4. Interestingly we observed preferential co-precipitation of BRD4 with NIPBL (Fig. 1c). The opposite approach, i.e., precipitation of BET proteins and analysis of co-precipitated NIPBL led to the same results (Fig. 1c). We then analyzed co-precipitation of endogenous NIPBL following precipitation of FLAG-tagged wild type or truncated BRD4 (lacking the ET and CTD domains; BRD4∆C), which were expressed in HEK293T cells. Results showed the absence of NIPBL interaction when using the truncated BRD4∆C protein (Fig. 1d). Finally, interaction of N-terminal NIPBL with BRD4 ET domain was assessed in an in vitro pull-down experiment with purified proteins, which confirmed direct interaction (Fig. 1e).

Thus, we have revealed a direct interaction of NIPBL with BET proteins, and we have been able to define the interaction surfaces. While the NIPBL interaction in the yeast two-hybrid system was not restricted to a specific BET member, preferential interaction with BRD4 was observed in mammal cells.

### Transcriptome analysis upon *Nipbl*, *Brd2*, and *Brd4* knockdown reveals significant overlapping in genes regulated by NIPBL and BRD4

**Fig. 2:**
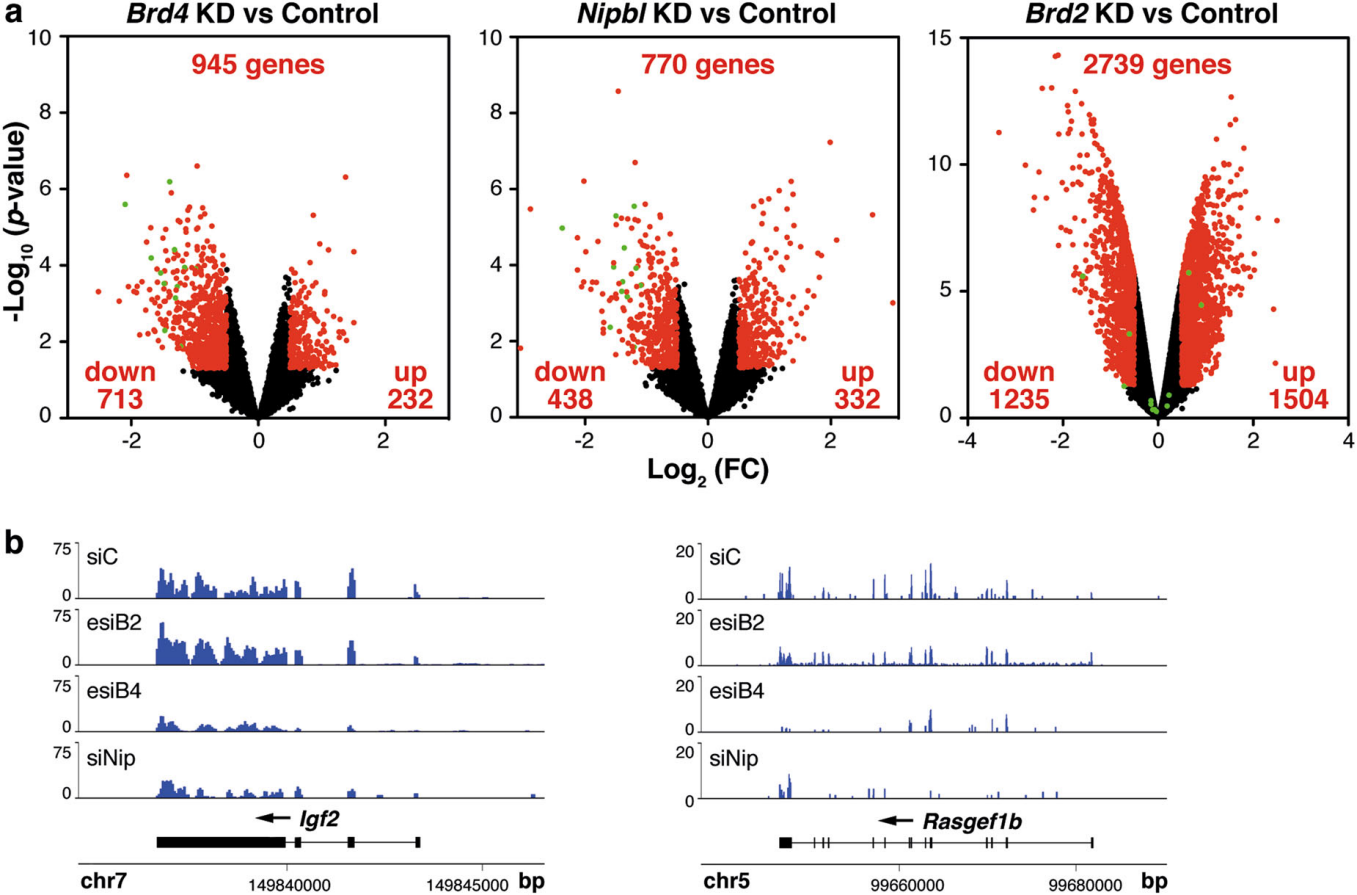
RNA-Seq analysis of P19 cells reveals changes in gene expression upon depletion of NIPBL, BRD2, or BRD4. **a** Volcano plots of misregulated genes upon knocking down (KD) of the indicated genes. Established *p*-value and fold change (FC) cutoff is indicated by black-red colors. Numbers inside plots indicate total number of misregulated genes and number of downregulated or upregulated genes. Genes selected for validation are indicated in green. **b** Expression profiles of two genes in control and depleted cells, as indicated, were selected as examples. siC, Control siRNA; esiB2, *Brd2* esiRNA; esiB4, *Brd4* esiRNA; siNip, *Nipbl* siRNA. Numbers in *y*-axis denote expression levels while numbers in *x*-axis indicate genome positions

**Fig. 3:**
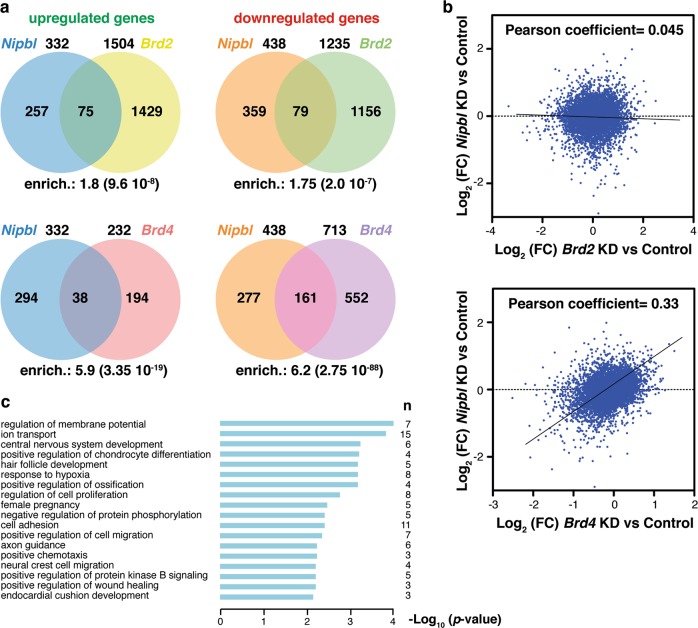
RNA-Seq analysis uncovers a high overlapping of misregulated genes upon NIPBL and BRD4 depletion. **a** Venn diagrams representing overlapping of misregulated genes upon knocking down of the indicated genes. Numbers on the diagrams indicate the total number of misregulated genes in each condition. Enrichment (enrich.) with the associated *p*-value between brackets, as determined by hypergeometric test, is also indicated for the different comparisons. **b** Pearson correlation of altered gene expression after *Nipbl* knockdown, compared with *Brd4* and *Brd2* knockdowns. FC fold change. **c** Gene ontology (GO) analysis of commonly downregulated genes upon NIPBL and BRD4 depletion, showing different categories of biological processes. *n* indicates the number of genes in the different categories

Since NIPBL has been independently associated both with BRD2 and with BRD4, we decided to compare the transcriptome of cells depleted of NIPBL, BRD2, or BRD4 with the aim of identifying preferential functional BET partners of NIPBL. For our analyses, we chose the P19 murine pluripotent embryonic carcinoma cell line because such cells have been widely used in developmental studies and are easy to manipulate and to transfect^37^. A commercially available and previously validated esiRNA^38^ was used to knockdown *Brd2*, while commercially available esiRNA and siRNA molecules were used for *Brd4* and *Nipbl* knockdown, respectively. Then, the transcriptomes of the multiple knocked down cells were analyzed in duplicate by RNA-Seq. We selected mild-knockdown conditions (supplementary Fig. S1) to mimic heterozygous mutations in CdLS. Principal component analysis demonstrated strong similarity between replicates and clearly separated different knockdown samples (supplementary Fig. S2). Given our moderate knockdown conditions we decided to take into account changes in gene expression with a |Log_2_ (fold changes)| ≥ 0.5. Thus, RNA-Seq analysis with this value and an established *p*-value < 0.05 identified 770, 2739, and 945 genes differentially expressed upon *Nipbl*, *Brd2*, or *Brd4* downregulation, respectively (Fig. 2, supplementary Table S1). Interestingly, a strong overlap between the set of genes misregulated upon *Nipbl* or *Brd4* downregulation was observed (5.9-fold enrichment for upregulated genes, *p* = 3.35 × 10^−19^ and 6.2-fold enrichment for downregulated genes, *p* = 2.75 × 10^−88^) (Fig. 3a). In fact, changes in gene expression upon *Nipbl* knockdown highly correlated with changes in gene expression upon *Brd4* knockdown (Pearson Correlation coefficient = 0.33, *n* = 12,000 active genes), but not with those observed upon *Brd2* knockdown (Pearson Correlation coefficient = 0.045, *n* = 12,000 active genes) (Fig. 3b). Two examples of genes commonly downregulated after *Brd4* or *Nipbl* knockdown, but not upon *Brd2* knockdown are shown in Fig. 2b. Therefore, among NIPBL-dependent genes, a significant set shows dependence on BRD4 but not on BRD2. We did not observe alterations in *Nipbl* expression when decreasing BRD4 protein (fold change = 1.002) or vice versa (fold change = 0.997), indicating that the effects of each knockdown are not due to changes in the expression of its counterpart.

Gene ontology (GO) analysis indicated that NIPBL-regulated genes shared more categories with BRD4-regulated genes than with BRD2-regulated genes (supplementary Fig. S3). Interestingly, GO analysis of common downregulated genes upon NIPBL or BRD4 depletion showed enrichment in categories such as ion transport, central nervous system development, chondrocyte differentiation, regulation of ossification, hair follicle development, axon guidance, and cell proliferation (Fig. 3c), which are potentially related to many of the observed traits of CdLS probands (i. e. psychomotor delay and intellectual disability, craniofacial abnormalities, skeletal deformations, hirsutism, and other developmental alterations)^31^.

Out of this set of co-regulated genes, we selected 12 of them (*Adamts17*, *Ajap1*, *Chst1*, *Clstn2*, *Crybg1*, *Dner*, *Igf2*, *Kcnc1*, *Kcnk3*, *Rasgef1b*, *Scml2*, and *Zbtb16*) representative of the indicated GO categories, for validation of the RNA-Seq results by quantitative PCR (qPCR) (Fig. 2a, green dots in volcano plots). In each case, both BRD4 and NIPBL depletion led to downregulation of gene expression, while BRD2 depletion resulted in variable effect with unaltered expression of most of the analyzed genes (supplementary Fig. S4), confirming the RNA-Seq data. Additional genes (*Nog*, *Pim2*, and *T*), not included in the commonly regulated set, were also tested as controls. As expected, qPCR analysis was in agreement with the RNA-Seq data (supplementary Fig. S4). As the knockdown of *Brd4* and *Nipbl* was not previously assessed in P19 cells, their transcriptional effect on such genes was further confirmed using additional siRNA molecules (supplementary Figs. S1 and S4).

Taken together, these data strongly indicate that BRD4 and NIPBL have a large number of common target genes, which are related to biological processes potentially linked to CdLS phenotype.

### NIPBL and BRD4 stabilize each other at promoters of co-regulated genes

**Fig. 4:**
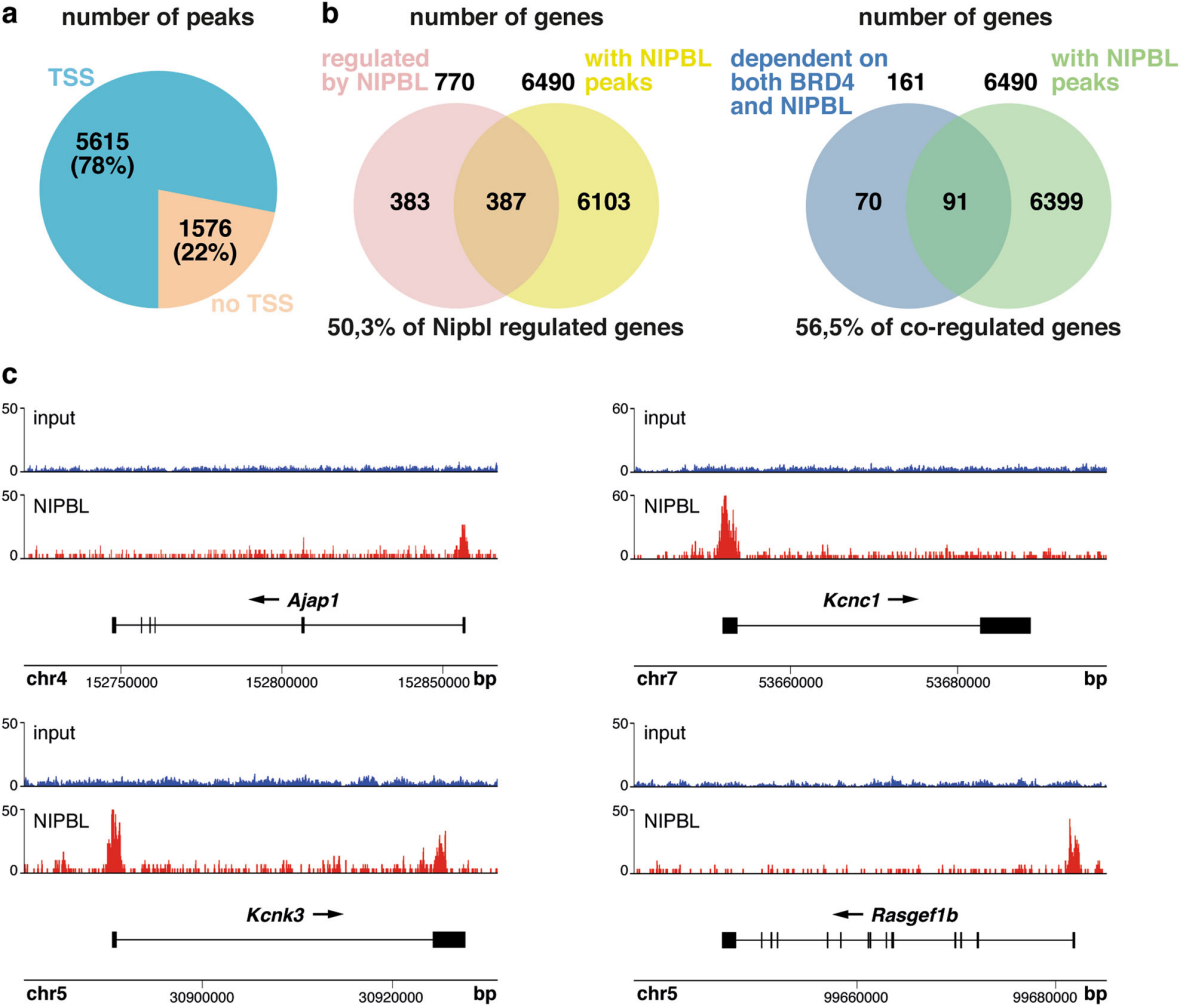
ChIP-Seq analysis shows major NIPBL localization at promoter regions. **a** Sectors diagram indicating the percentage of NIPBL peaks, as determined through ChIP-Seq analysis, at transcription start sites (TSS) or out of TSS (no TSS). **b** Venn diagrams showing the number of genes with NIPBL peaks overlapping either with genes regulated by NIPBL or with genes commonly downregulated upon NIPBL and BRD4 depletion, i.e., genes dependent on both proteins. Numbers on the diagrams indicate the total number of genes in each condition. Percentage of overlapping genes, referred to the total number of regulated genes in each analysis is also shown. **c** NIPBL localization on genes selected for further studies is shown. *y*-axis denotes ChIP signal amplitude, while *x*-axis indicates genome positions

Being *NIPBL* the most frequent CdLS causative gene, and because it has not been clearly established if it preferentially localizes at promoters or enhancers^33,34,36,39^, we decided to conduct a ChIP-Seq analysis on P19 cells. We identified 7191 NIPBL peaks corresponding to 6490 genes. Of the total number of peaks, 5615 mapped to promoter (TSS) regions (Fig. 4a; supplementary Table S2), strongly associating NIPBL to promoters rather than to enhancers.

Among the 770 genes that were misregulated upon *Nipbl* knockdown, 387 (50.3%) presented NIPBL peaks (Fig. 4b) (1.7-fold enrichment, *p* = 3.3 × 10^−33^). Among the 161 genes commonly downregulated when knocking down *Nipbl* or *Brd4*, 91 (56.5%) presented NIPBL peaks at the promoter region (1.9-fold enrichment, *p* = 1.6 × 10^−12^) (Fig. 4b). We selected some of these genes (Fig. 4c) to analyze NIPBL and BRD4 occupancy at promoters following depletion of each protein. In order to assess the importance of the NIPBL–BRD4 interaction in the context of chromatin association and gene expression, we also managed to analyze the effects of a truncated BRD4, which lacks the C-terminal region containing the ET domain interacting with NIPBL. As shown in Fig. 1d, the BRD4∆C construct proved unable to interact with NIPBL. However, it is expected that it still binds to the chromatin as it retains the bromodomains and the mB.

**Fig. 5:**
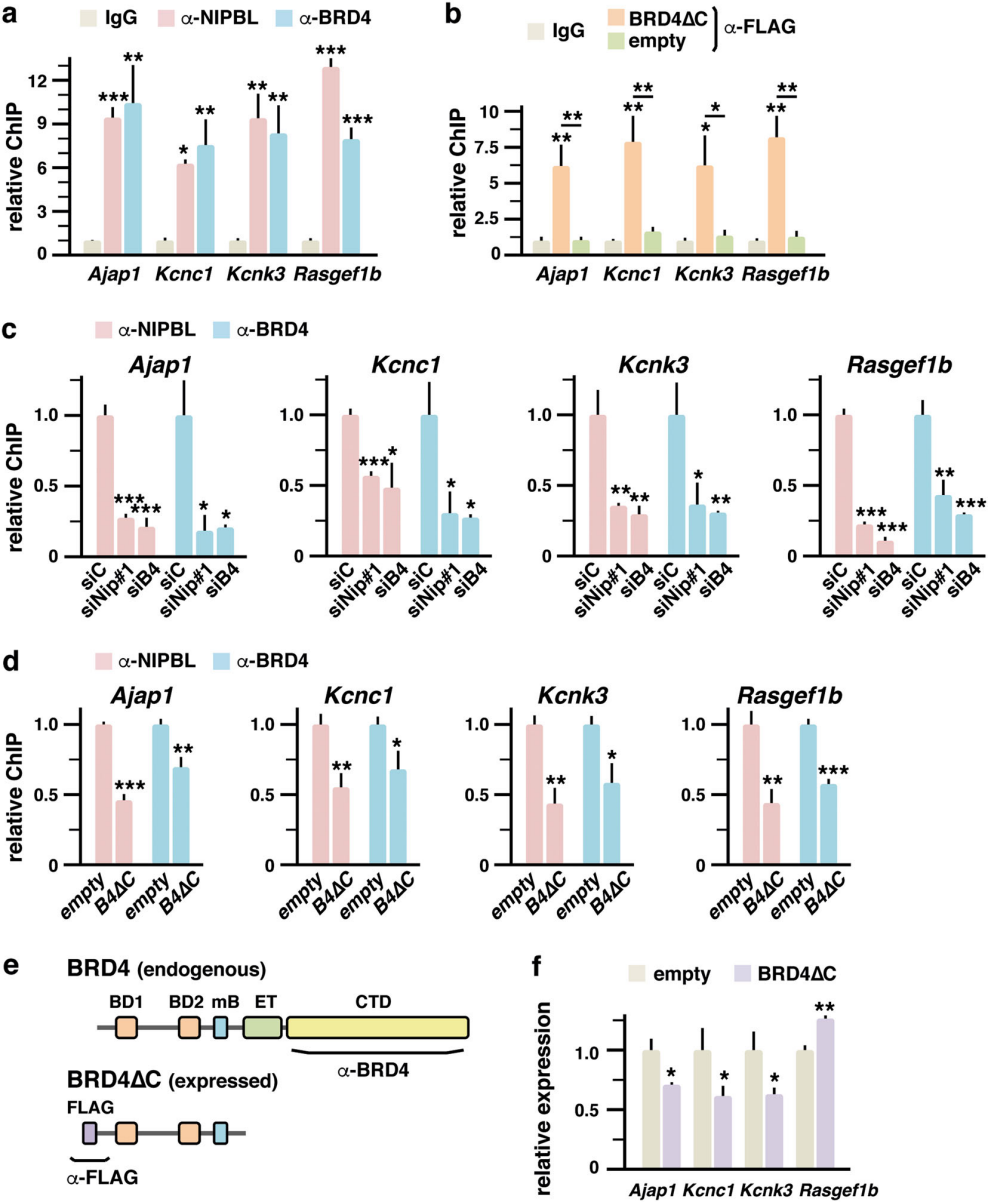
NIPBL and BRD4 stabilize each other on the chromatin. **a** Localization of NIPBL and BRD4 at the promoters of the indicated genes was assessed through ChIP analysis with the indicated antibodies (α-) in comparison with normal rabbit IgG. Relative ChIP levels are represented. **b** Localization of BRD4∆C at the promoters of the indicated genes was assessed through ChIP analysis with FLAG antibodies in comparison with normal mouse IgG. FLAG signal was determined in cells transfected either with the expression construct for the BRD4∆C protein as with empty vector. Relative ChIP levels are represented. **c** NIPBL and BRD4 localization at the promoters of the indicated genes was assessed through ChIP analysis after *Nipbl* or *Brd4* knockdown (siNip#1, *Nipbl* siRNA #1, and siB4, *Brd4* siRNA, respectively) in comparison with control conditions (siC, Control siRNA). Relative ChIP levels are represented. **d** NIPBL and BRD4 localization at the promoters of the indicated genes was assessed through ChIP analysis after transfection of the BRD4∆C expression construct (B4∆C) in comparison with control transfections with empty vector. Relative ChIP levels are represented. **e** Schematic representation of the endogenous wild-type BRD4 and the expressed truncated BRD4∆C protein, indicating the regions recognized by the antibodies (α-) used for ChIP analysis. **f** Expression levels of the indicated genes, after transfection of the BRD4∆C expression construct were determined by qPCR. Relative levels of expression are represented. Cells transfected with empty vector were used as control. Values are means ± s.d. from three independent experiments analyzed in triplicate. Statistical significance of differences between the different conditions and IgG (**a**, **b**), control siRNA (siC) (**c**), or empty vector (**d**, **f**), is indicated on top of each bar. Statistical significance of differences between other conditions is also indicated with a line. Significance was analyzed by Student’s *t* test (*p < 0.05, **p < 0.01, ***p < 0.001)

We first analyzed ChIP signal of NIPBL and BRD4 antibodies on the selected promoters in comparison with normal rabbit IgG. As shown in Fig. 5a, results confirmed the presence of the proteins at the selected promoters. We also verified localization of the expressed FLAG-tagged BRD4∆C protein at these promoters by using FLAG antibodies (Fig. 5b). This experiment confirmed our prediction that binding of the truncated protein to the chromatin is preserved. Then, we monitored the effect of knocking down *Nipbl* or *Brd4* on the localization of endogenous NIPBL and BRD4 at promoters. Knockdown experiments showed mutual dependence for chromatin occupancy at selected promoters (Fig. 5c). Next, we analyzed the effect of expressing truncated BRD4. Expression of BRD4∆C was able to displace the endogenous BRD4 from the analyzed promoters (Fig. 5d). Since commercial BRD4 antibodies were raised against the BRD4 CTD domain, they only recognize the endogenous wild-type protein and not truncated BRD4 (Fig. 5e). More importantly, the BRD4∆C construct led to dissociation of NIPBL from the chromatin (Fig. 5d). In addition, the effects of truncated BRD4 on promoter localization of endogenous BRD4 and NIPBL were accompanied by altered expression of the corresponding genes (Fig. 5f).

Thus, our results indicate that NIPBL and BRD4 stabilize each other on the chromatin in an interaction-dependent manner, suggesting functional cooperation of NIPBL and BRD4.

### NIPBL–BRD4 interplay in human fibroblasts and *Drosophila melanogaster*

**Fig. 6:**
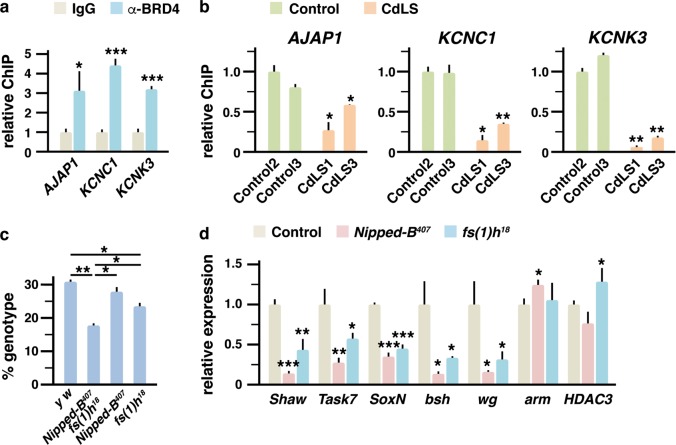
NIPBL–BRD4 relationship in different cellular models. **a** Localization of BRD4 at the promoters of the indicated genes was assessed in human fibroblasts from Control2 healthy donor (see “Materials and methods”) through ChIP analysis with BRD4 antibody (α-BRD4) in comparison with normal rabbit IgG. Relative ChIP levels for each gene were represented. Values are means ± s.d. from three independent experiments analyzed in triplicate. **b** BRD4 localization at the promoters of the indicated genes was assessed through ChIP analysis in fibroblasts from two healthy donors (Control2 and Control3) or from two CdLS probands with *NIPBL* mutations (CdLS1 and CdLS3). Relative ChIP levels for each gene were represented. Values are means ± s.d. from two independent experiments analyzed in triplicate. **c** Quantification of the proportion of the different genotypes in the female progeny from flies crosses between *Nipped-B*^*407*^ males and *fs(1)h*^*18*^ females. Values are means ± s.d. Data correspond to the analysis of 107 individuals from two independent experiments. **d** Expression levels of the indicated genes as determined in single heterozygous *Nipped-B*^*407*^ and *fs(1)h*^*18*^ flies and in y w controls, were determined by qPCR. Relative levels of expression are represented. Statistical significance of differences between the various conditions and IgG (**a**), control2 (**b**), or y w control (**d**) is indicated on top of each bar. Statistical significance of differences between other conditions is indicated with a line. Significance was analyzed by Student’s *t* test (*p < 0.05, **p < 0.01, ***p < 0.001)

We next analyzed additional models to study the relation between NIPBL and BRD4. In this context, we decided to assess whether BRD4 occupancy at NIPBL–BRD4 co-regulated genes was altered also in human CdLS probands carrying *NIPBL* mutations. To this end, we cultured fibroblasts from healthy and CdLS individuals^40^ and assessed BRD4 occupancy at the selected promoters. We first employed BRD4 antibodies on human samples relative to normal rabbit IgG, revealing the presence of BRD4 at selected promoters (Fig. 6a). Notably, ChIP experiments also led us to confirm reduced occupancy of BRD4 at promoters in human NIPBL-defective cells (Fig. 6b).

Finally, to evaluate in vivo the interaction between NIPBL and BRD4, we exploited existing mutants of *Drosophila*. In this organism, *Nipped-B* codes for NIPBL while *fs(1)h* codes for a unique BET homolog. Upon mating of *fs(1)h*^*18*^ and *Nipped-B*^*407*^ heterozygotes, we scored the number of trans-heterozygous females in the F1 progeny. Males hemizygous for *fs(1)h*^*18*^ die during larval stages, thus trans-heterozygous F1 males could not be reliably counted^41^. However, the observed percentage of trans-heterozygous females was 17.8% (Fig. 6c), revealing significant reduction from expected mendelian inheritance (25%). We also analyzed expression of selected genes in single heterozygous *Nipped-B*^*407*^ and *fs(1)h*^*18*^ flies and in controls. Such analysis indicated that expression of *Shaw* and *Task7*, respectively the *Drosophila* orthologs of *Kcnc1* and *Kcnk3*, was also dependent on both NIPBL and BET. Other genes such as *SoxN*, *bsh*, and *wg*, the fly orthologs of *Sox2*, *Nanog*, and *Wnt1*, also showed codependence on NIPBL and BET proteins, while, as controls, *arm* and *HDAC3*, the orthologs of *Ctnnb1* and *Hdac8*, were differently affected in the two mutant lines (Fig. 6d).

Overall, our results establish a functional link between NIPBL and BRD4, which is present in human fibroblasts and *D. melanogaster*.

## Discussion

In this work we report a novel interaction-mediated cooperation between NIPBL and BRD4 to control transcription of a large set of genes. Such cooperation is supported by a number of findings: (i) NIPBL directly interacts with BRD4 via its ET domain, (ii) transcriptome analysis indicates that NIPBL and BRD4 regulate a common set of genes, (iii) ChIP experiments strongly suggest that NIPBL and BRD4 co-localize and stabilize each other at promoters of co-regulated genes, and (iv) a dominant negative form of BRD4, which lacks the NIPBL interacting domain and retains ability to bind the chromatin, is able to displace endogenous BRD4 and NIPBL from promoters with consequences in gene expression regulation.

Although co-precipitation of NIPBL and BRD4 has been recently described^32^, direct interaction was not reported so far, and the interacting domains were not known. Concerning this, the ET domain represents an essential domain for BET function, a finding supported by previously reported interactions^19^. Since the dominant negative BRD4 molecule causes BRD4 and NIPBL to detach from the chromatin and alters gene expression, the NIPBL–BRD4 interaction seems relevant for cooperative transcriptional control. We show that NIPBL interacts with multiple BET proteins by yeast two-hybrid. Interestingly, the interaction with BRD2 was not confirmed in mammal cells. However, we cannot exclude NIPBL association with different BET members depending on the physiological conditions or cellular contexts. In this regard, although some of the results obtained in P19 cells have also been confirmed in human fibroblasts and in *Drosophila*, it would be of interest to extend our studies to additional cellular models.

Our RNA-Seq analyses indicate that NIPBL shares a large number of common target genes with BRD4 but not with BRD2, suggesting the possibility that NIPBL and BRD4 cooperate in gene regulation. Despite this, NIPBL, as part of the CTCF-cohesin complex, has been associated with BRD2 in the context of enhancer assembly^30^. On the other hand, BRD2 has also been associated with CTCF, but not specifically with NIPBL, in the context of transcription barriers along chromatin^42^. Animal models of CdLS have revealed robust cohesion all along the chromosomes and unaltered DNA replication, repair, and chromosome segregation. However, models display local impairment of cohesin loading on certain promoters, which correlates with gene dysregulation, suggesting that CdLS-associated phenotype derives from altered transcription rather than from defective chromosomal cohesion processes^35^. Moreover, an important role of NIPBL in chromatin architecture and transcription has been decoupled from cohesin and CTCF^36,43^. Besides this, it has been well reported that removal of cohesin does not cause major changes in gene expression^44–46^. Thus, it is plausible that NIPBL displays general structural and architectural roles on the chromatin as a component of the cohesin complex, also involving BRD2, but a tighter relation is established with BRD4 for specific control of gene expression. In relation to this, our GO analysis showed enrichment in a number of categories related with many of the observed traits in CdLS probands^31^, a finding that might help explain the recent observation of CdLS-like defects in individuals carrying mutations in *BRD4*^32^.

Our ChIP-Seq data reveal preferential association of NIPBL with promoters, while BRD4 has been localized at both promoters and enhancers, with a relevant role in superenhancers^13,47–50^. NIPBL localization at promoters has been previously reported^36,51^. Considering this, it would be of interest to address whether interaction between enhancer-associated BRD4 and promoter-associated NIPBL might participate in promoter–enhancer assembly. Provided that most common misregulated genes under knockdown conditions are related to important developmental processes, these hypothetical promoter–enhancer contacts acquire special importance at relevant developmental regulated enhancers. Thus, major localization of NIPBL at promoters does not exclude enhancer-mediated roles of NIPBL, due in part to putative enhancer–promoter contacts but also to direct localization of NIPBL at certain enhancers.

Our analysis also reveals that a significant proportion of NIPBL bound genes appear unaffected by NIPBL knockdown or fall out of the set of commonly regulated genes. Although our moderate knockdown conditions may have excluded a number of genes from the set of affected genes, it is also possible that NIPBL location at some regions is unrelated to gene expression, but is related, for example, to its cohesin-loading role. In this sense we have previously mentioned the reported absence of major transcriptional changes upon removal of cohesin^44–46^. We also assume the concurrence of indirect effects on gene expression by NIPBL depletion and regulation of particular genes by NIPBL independently of BRD4.

ChIP experiments indicate that NIPBL and BRD4 mutually stabilize on the chromatin. We show such phenomenon in both mouse and human cells. The effect of truncated BRD4 as a dominant negative molecule able to displace both endogenous BRD4 and NIPBL from the chromatin strongly supports this idea. Mutual stabilization of NIPBL and BRD4 on the chromatin prompts the question of whether these proteins form a complex before chromatin binding or whether one of them is previously recruited to the chromatin priming the association to its partner. In this context, it is tempting to speculate that BRD4, which is able to recognize acetylated histones, could initially associate to the chromatin to subsequently recruit NIPBL. However, Olley et al.^32^ describe that more typical CdLS was observed with a de novo missense mutation in the 2nd bromodomain of BRD4 altering its chromatin binding capacity but not the ability to interact with NIPBL, which is in agreement with our observation of the ET domain mediating the interaction. This suggests that in patients with this BRD4 mutation, a complex with NIPBL is formed, but according to our results, unable to stabilize at the chromatin. Further research on this matter will clarify on sequential mechanisms involved in the cooperative action of both proteins.

Finally, the analysis of *Drosophila* as an animal model revealed the existence of a genetic interaction between *Nipped-B* and *fs(1)h*, the fly genes coding for NIPBL and the only BET protein, which is based on altered mendelian ratios. While this manuscript was in preparation, using a different *fs(1)h* mutant allele, Pherson et al.^51^ also reported genetic interaction of *Nipped-B* and *fs(1)h* in *Drosophila* in the context of cohesin occupancy of origin of replication chromatin, supporting our results. Gene expression analysis of selected genes in single mutants also confirmed co-regulation in *Drosophila*. Of special interest were *Shaw* and *Task7*, the orthologs of *Kcnc1* and *Kcnk3*, which were downregulated in both single mutants. In addition, *wg*, the ortholog of mouse *Wnt1*, also showed altered expression in both single mutants, in agreement with previously reported *wnt1* misregulation in zebrafish after *nipblb* downregulation^40^.

In conclusion, we provide evidence for direct interaction and cooperation of NIPBL and BRD4 to control gene expression, which could be relevant to CdLS-like traits of probands with mutations in the corresponding genes^31,32^. In particular, our study establishes for the first time a functional basis for the association of these proteins. Further investigation of such interaction will provide a detailed molecular description of gene expression in an important rare human disease.

## Materials and methods

### Yeast two-hybrid

Yeast manipulation and ß-galactosidase assays for yeast two-hybrid experiments were performed with the DUALhybrid Kit (DualSystems Biotech, Schlieren, Zurich, Switzerland) using the pLexA-N bait and pGAD-HA prey vectors, according to the manufacturer instructions and as previously described^18^. pLexA-BRD2, pLexA-BRD3, pLexA-BRD4, pLexA-mB, and pLexA-ET constructs were described previously^18,38^.

### Plasmid constructs and siRNA molecules

Expression construct for full-length mouse *Brd4* has been previously described^18^ and was based on pAdRSV-Sp vector with an N-terminal FLAG tag. Deletion of the C-terminal half of BRD4 (amino acids 603–1400) in the BRD4ΔC construct was generated by standard PCR techniques. GST-ET and GST-FLAG-NIPBL, generated by standard PCR techniques, were based on pGEX-6P-3 vector (GE Healthcare, Buckinghamshire, UK) and containing amino acids 609–718 of BRD4 and 212 to 449 of NIPBL, respectively. The siRNA molecules used for the different knockdowns were all obtained from Sigma-Aldrich (St. Louis, MO, USA), and are as follows: *Nipbl* siRNA #1, GCGAUAUACCCGUCUUGUU (SASI_Mm02_00351489); *Nipbl* siRNA #2, GGAAGAUUGGUAGCUUGUA (SASI_Mm02_00351487); *Brd4* siRNA, GAGAAGGACAAGAAGGAAA; Control siRNA, CGUACGCGGAAUACUUCGA; *Brd4* esiRNA, MISSION esiRNA EMU051511; *Brd2* esiRNA, MISSION esiRNA EMU067621; Control esiRNA, MISSION esiRNA EHUFLUC. Control siRNA and esiRNA correspond to the *Luciferase* gene.

### Cell culture and transfection

Human HEK293T cells were cultured in Dulbecco’s Modified Eagle’s Medium (Sigma-Aldrich) supplemented with 10% fetal bovine serum (Sigma-Aldrich). This line was used for IP experiments. Mouse P19 cells were directly purchased from ATCC (LGC Standards, Barcelona, Spain) as authenticated and were cultured in α-modified Minimum Essential Medium (HyClone, Logan, UT, USA) supplemented with 7.5% calf serum (HyClone) and 2.5% fetal bovine serum. Transfections were performed with Lipofectamine 2000 or Lipofectamine RNAiMax (Invitrogen, Life Technologies, Paisley, UK) for plasmids (24 h) and siRNAs (72 h), respectively.

### Human fibroblasts

Human fibroblasts were raised from biopsies from two CdLS patients with known mutation in *NIPBL* and two age-matched healthy controls described by Pistocchi et al. ^40^. Patients with CdLS, assessed with severe mental retardation, correspond to CdLS1 and CdLS3 individuals described by Pistocchi et al.^40^. Healthy donors, one male and one female Italian pediatric patients, were undergoing surgical procedures for dermatological testing, and correspond to Control2 and Control3 described by Pistocchi et al.^40^. Fibroblasts were cultured in RPMI medium supplemented with 10% FBS, and samples from the same passage (p2) were used. Experiments conformed to the principles set out in the WMA Declaration of Helsinki and in the Belmont Report.

### *D. melanogaster* culture

Flies were maintained on cornmeal, yeast, and agar/molasses medium at 25 °C according to standard protocols. We used lines with the *fs(1)h*^*18*^ mutant allele (Bloomington Stock #5285: fs(1)h^18^/FM7c) and the *Nipped-B*^*407*^ allele (y w; Nipped-B^407^ P{w+}57B/CyO, Kr-GFP)^52^, a loss of function allele. *Drosophila Nipped-B* mutants are recognized as a model of CdLS (Flybase: FBhh0000605). In all experiments, mutant and control flies were grown at the same time with the same batch of food preparation. Two biological repetitions of the experiment were performed in triplicate. The cross to evaluate interaction between *fs(1)h* and *nipped-B* was done using the following genotypes: ten virgin females (fs(1)h^18^/FM7c) and five males (y w; Nipped-B^407^ P{w+}57B/CyO, Kr-GFP). The different genotypes were collected looking at the different flies’ phenotypes. Flies carrying *fs(1)h*^*18*^ allele did not display bar eyes; while flies carrying *Nipped-B*^*407*^ allele did not display curly wings.

### Protein IP, immunoblotting, purification, and in vitro pull-down

For IP, cells were extracted with buffer [150 mM NaCl, 50 mM Tris-HCl pH 7.5, 2.5 mM Mg^+2^, 1% Triton X-100, and complete protease inhibitor cocktail w/o EDTA (Roche, Mannheim, Germany)] and incubated for 1 h at 37 °C with Bit-nuclease (bimake.com, Munich, Germany). Concentration was determined by the Bradford reactive assay (Bio-Rad, Hercules, CA, USA) and 1 mg of protein was incubated overnight at 4 °C in rotation with FLAG-beads (Sigma-Aldrich) or the corresponding antibody. Antibodies were precipitated after 2 h of incubation at 4 °C in rotation with protein A Dynabeads (ThermoFisher Scientific, Waltham, MA, USA). After washing, proteins were eluted from beads with 20 µL of Laemmli buffer and 10 min of boiling before the analysis by immunoblotting. For this, eluted proteins or whole extracts (25 µg of protein) were separated in an SDS gel and subsequently transferred to a PVDF membrane (GE Healthcare) and blotted with antibodies. The membrane was processed with a chemiluminescence ECL system (Bio-Rad) and monitored in a ChemidDoc XRS apparatus (BioRad). The antibodies used were: mouse anti-FLAG M2 (1:2000, Sigma-Aldrich), mouse anti-α-TUBULIN (1:5000, Sigma-Aldrich), rabbit anti-NIPBL (1:3000, A301-779A, Bethyl Laboratories, Inc., Montgomery, TX, USA), rabbit anti-BRD4 (1:2000, A301-985A100, Bethyl Laboratories, Inc.) rabbit anti-BRD2^18^ (1:1000) and horseradish peroxidase (HRP)-conjugated goat anti-mouse IgG and goat anti-rabbit IgG (1:10000, Sigma-Aldrich). Normal rabbit IgG (Sigma-Aldrich) was used as a negative control. GST, GST-ET, and GST-FLAG-NIPBL proteins were produced in *Escherichia coli* DH5α and purified on Glutathione Sepharose beads (GE Healthcare). While GST and GST-ET proteins were kept bound to beads, FLAG-NIPBL was excised from GST by using the PreScission Protease (GE Healthcare) according to manufacturer’s instructions. In vitro pull-down assays were performed as previously described^53^.

### RNA extraction, quantitative real-time PCR (qPCR), and RNA-Seq

Total RNA was extracted from P19 cells using the NZY Total RNA isolation kit (NZYTech, Lisbon, Portugal) and was retro-transcribed using the iScript cDNA Synthesis kit (BioRad). qPCR was performed with Power SYBR Green (Applied Biosystems, Carlsbad, CA, USA) in the ViiA7 Real-Time PCR System (Applied Biosystems). The *RpLp0* gene was used as a reference gene to analyse relative expression. For analysis of gene expression in *D. melanogaster*, total RNA was extracted from whole animals of each of the three genotypes (*n* = 15) using the RNeasy mini kit (Qiagen). Two micrograms of RNA was retro-transcribed using the SuperScript™ VILO™ cDNA Synthesis Kit (Invitrogen) and qPCR was performed with SsoFast™ EvaGreen^®^ Supermix (BioRad) using the CFX96™ Real-Time PCR Detection Systems (BioRad). Amplification of *RpL32* transcripts was used as a normalizer. Normalization was done according to ref. ^54^. The primers used are detailed in supplementary Table S3. For RNA-Seq, total RNA was extracted using the RNeasy kit (QIAGEN, Austin, TX, USA) and analyzed in CABIMER Genomics facility. The libraries were prepared with the TruSeq Stranded TOTAL RNA kit (Illumina, San Diego, CA, USA) and the sequencing was performed with the NextSeq500 HIGH-Output and 1x75bp length parameters. RNA-Seq data were primarily filtered using the FASTQ Toolkit v1.0.0 program. Data were aligned using Subjunc function from Rsubread^55^ v.1.28.1 bioconductor package, to map reads to the mm9 mouse reference genome, using TH1 = 2 and unique = TRUE parameters. The downstream analysis was performed on bam files with duplicates removed using the samtools^56^ v.0.1.19 rmdup command. FeatureCounts() function from Rsubread v.1.28.1 bioconductor package was used to assign reads to UCSC mm9 genes using GTF.featureType = “exon” and GTF.attrType = “gene_id” parameters on duplicate removed bam files. Then differential gene expression analysis was performed using the voom/limma^57^ v.3.34.9 and edgeR^58,59^ v.3.20.9 bioconductor packages. Genes that were expressed at >1 counts per million mapped reads in ≥2 replicates were analyzed. CalcNormFactors() function using TMM method was used to normalize samples. For each comparison (si*Nipbl*#1 vs siControl, esi*Brd4* vs siControl, and esi*Brd2* vs siControl), we selected those genes that were upregulated or downregulated with a *p*-value < 0.05 and |log_2_(FC)| ≥ 0.5. Data have been deposited under accession number GEO: GSE132785.

### ChIP and ChIP-Seq

Cells were crosslinked in 1% formaldehyde for 10 min at room temperature followed by addition of glycine (125 mM as final concentration) for 5 min. Nuclei were isolated using lysis buffer 1 [5 mM Pipes pH 8, 85 mM KCl, 0.5% NP40, and complete protease inhibitor cocktail (Roche)] and were lysed using lysis buffer 2 [1% SDS, 10 mM EDTA, 50 mM Tris-HCl pH 8.1, and complete protease inhibitor cocktail (Roche)]. Chromatin was sheared into an average size of 500 bp by 8 or 20, 30 s pulses (30 s pause between pulses) for P19 cells and human fibroblasts, respectively at 4 °C in the water bath sonicator Bioruptor (Diagenode, Liège, Belgium). Thirty or eight micrograms of chromatin from P19 cells or human fibroblasts, respectively, was incubated overnight at 4 °C in rotation diluted 1:10 in IP buffer [0.01% SDS, 1.1% Triton X-100, 1.2 mM EDTA, 16.7 mM Tris-HCl pH 8.1, and 167 mM NaCl] and with the respective antibodies [3 µg of anti-BRD4 (A301-985A100, Bethyl Laboratories, Inc.), 3 µg of anti-NIPBL (A301-779A, Bethyl Laboratories, Inc.), 3 µg of anti-FLAG M2 (Sigma-Aldrich), or 3 µg normal rabbit or mouse IgG (Sigma-Aldrich) as negative controls]. IPs were incubated for 2 h at 4 °C in rotation with protein A or G Dynabeads (Invitrogen) for rabbit or mouse antibodies, respectively, and then washed with wash buffer 1 (0.1% SDS, 1% Triton X-100, 2 mM EDTA, 20 mM Tris-HCl pH 8.1, and 150 mM NaCl), wash buffer 2 (0.1% SDS, 1% Triton X-100, 2 mM EDTA, 20 mM Tris-HCl pH 8.1, and 500 mM NaCl), wash buffer 3 (0.25 M LiCl, 1% NP40, 1% sodium deoxycholate, 1 mM EDTA, and 10 mM Tris-HCl pH 8.1), and twice with TE buffer (10 mM Tris-HCl pH 8.0 and 1 mM EDTA). The complexes were eluted from the beads with elution buffer (1% SDS in TE buffer) by incubating twice 10 min at 65 °C. The eluates and the inputs (3 or 1 µg of chromatin from P19 cells or human fibroblasts, respectively) were incubated overnight at 65 °C for de-crosslinking. All the samples were treated with proteinase K (Roche) for 1 h at 37 °C and purified using the ChIP DNA Clean & Concentrator kit (Zymo Research, Irvine, CA, USA). ChIP-qPCR was performed with Power SYBR Green (Applied Biosystems) in the ViiA7 Real-Time PCR System (Applied Biosystems). The primers used are detailed in supplementary Table S3. ChIP-Seq was performed in GENECORE (EMBL Genomics Core Facility). The ChIP-Seq reads were aligned using align function from Rsubread^55^ v.1.28.1 bioconductor package, to map reads to the mm9 mouse reference genome, using TH1 = 2 and unique = TRUE parameters. The downstream analysis was performed on bam files with duplicates removed using the samtools^56^ v.0.1.19 rmdup command. MACS2^60^ version 2.1.1 was used to call NIPBL narrow peaks against input with a cutoff of −log10(*q-v*alue) = 15 and mfold = 5. To assess the overlapping between NIPBL peaks and TSS first we defined TSS as 2 kb windows (1 kb upstream and 1 kb downstream) for the TSS of all UCSC mm9 genes (knownGene), then bedtools^61^ v2.27.1 subtract command was used to determine those NIPBL peaks which overlap to TSS or not. AnnotatePeakInBatch() function from ChIPPeakAnno^62^ v3.10.1 bioconductor package was used to annotate peaks to UCSC mm9 genes. Data have been deposited under accession number GEO: GSE132784.

### Statistical analysis and additional tools

Statistical analyses were performed with the Prism 5.0a software (GraphPad). Data were generated from several repeats of different biological replicates. Mean values ± s.d. were represented in the different graphs. Except when indicated, data correspond to three independent experiments analyzed in triplicate. To determine significance of differences between conditions Student’s *t* tests for unpaired samples with confidence interval of 95% were computed. Significance between conditions were indicated with the symbols *p < 0.05, **p < 0.01, ***p < 0.001. Regression plots and determination of Pearson coefficients and *p*-value were performed using the Prism 5.0a software (GraphPad). To test the significance of overlapping in Venn diagrams, hypergeometric tests were performed in R, using the dhyper function from the Stats package. Venn diagrams were performed in Venny 2.1 (http://bioinfogp.cnb.csic.es/tools/venny/index.html). GO functional categories were analyzed using DAVID^63^.

## Supplementary information

Supplementary Figures

Supplementary table S1

Supplementary Table S2

Supplementary Table S3
