# Supplementary material for: The Cornelia de Lange Syndrome-associated factor NIPBL interacts with BRD4 ET domain for transcription control of a common set of genes
Source: Cell Death Dis. 2019 Jul 18;10(8):548. doi: 10.1038/s41419-019-1792-x (PMC6639259; doi:10.1038/s41419-019-1792-x)
Supplement: Supplementary file 1 — Supplementary Figures [file 41419_2019_1792_MOESM1_ESM.pdf]

The Cornelia de Lange Syndrome-associated factor NIPBL interacts with BRD4 ET domain for transcription control of a common set of genes

Noelia Luna-Peláez[1], Rosana March-Díaz[1], María Ceballos-Chávez[1], Jose A Guerrero-Martínez[1], Paolo Grazioli[2], Pablo García-Gutiérrez[1], Thomas Vaccari[3], Valentina Massa[2], Jose C Reyes[1], Mario García-Domínguez[1]*

[1]Andalusian Center for Molecular Biology and Regenerative Medicine-CABIMER
CSIC-Universidad de Sevilla-Universidad Pablo de Olavide.
Av. Américo Vespucio 24, 41092, Seville, Spain
[2]Dipartimento di Scienze della Salute, Università degli Studi di Milano, Via A. di Rudinì 8, 20142 Milano, Italy
[3]Dipartimento di Bioscienze, Università degli Studi di Milano, Via Giovanni Celoria 26, 20133 Milano, Italy

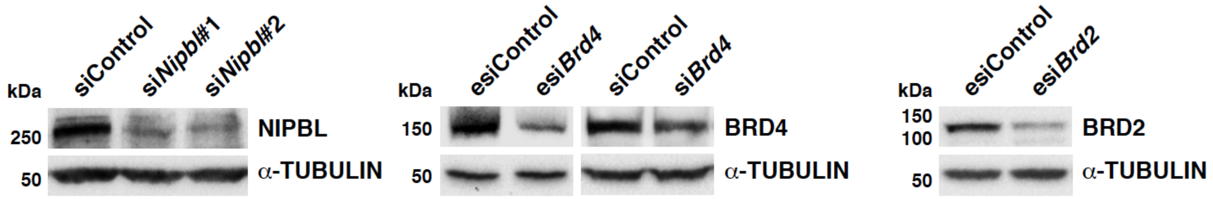

Supplementary Figure S1 Assessment of the knockdown of *Nipbl*, *Brd4* and *Brd2*. Analysis by western blot of protein depletion upon transfection of the indicated siRNA (si) or esiRNA (esi) molecules. Twenty micrograms of total protein were loaded per lane. α-TUBULIN was registered as a loading control.

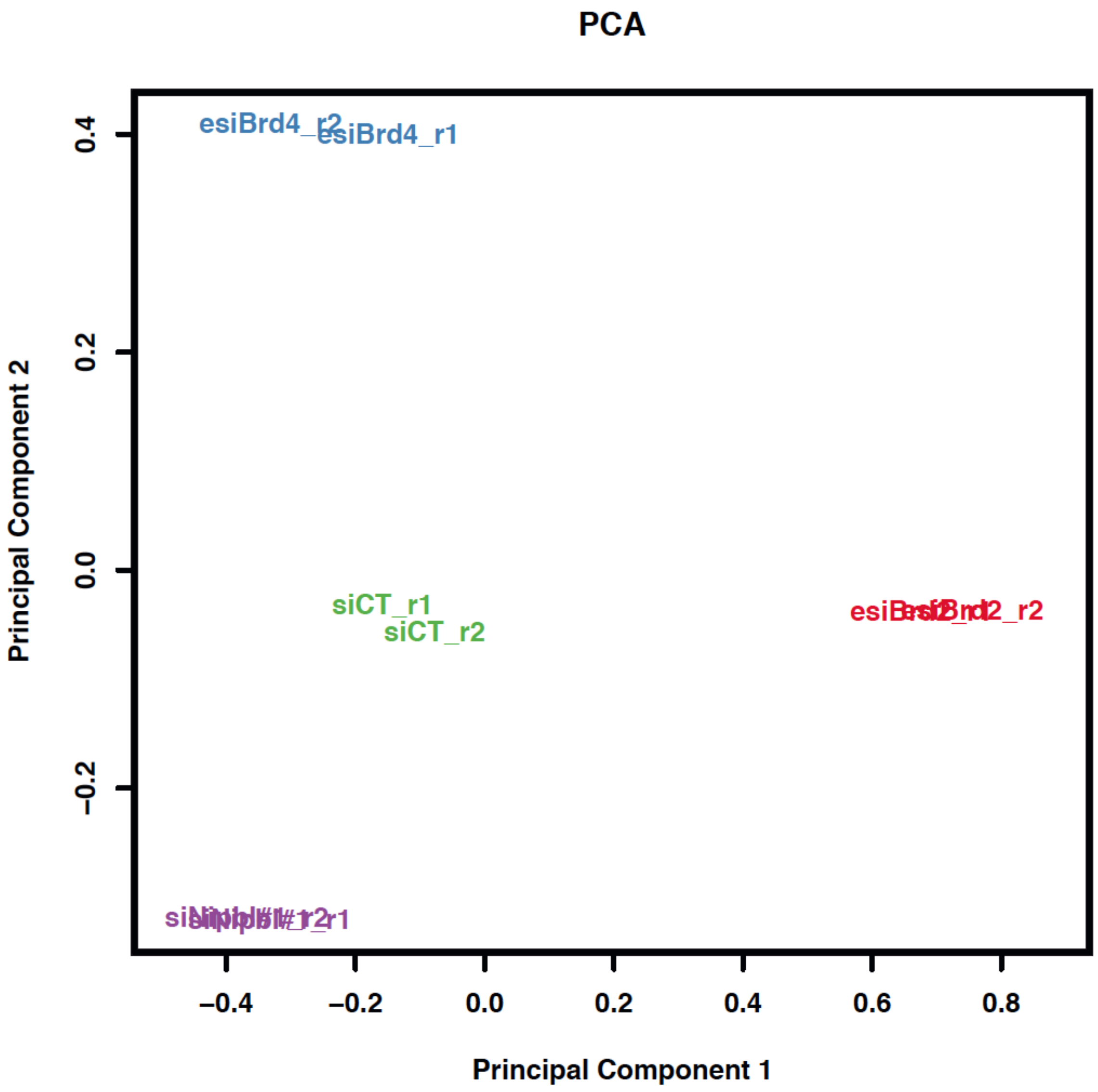

Supplementary Figure S2 Principal component analysis of RNA-Seq data. Principal component analysis (PCA) performed on TMM normalized RNA-seq expression data. Biological replicates are depicted in the same color.

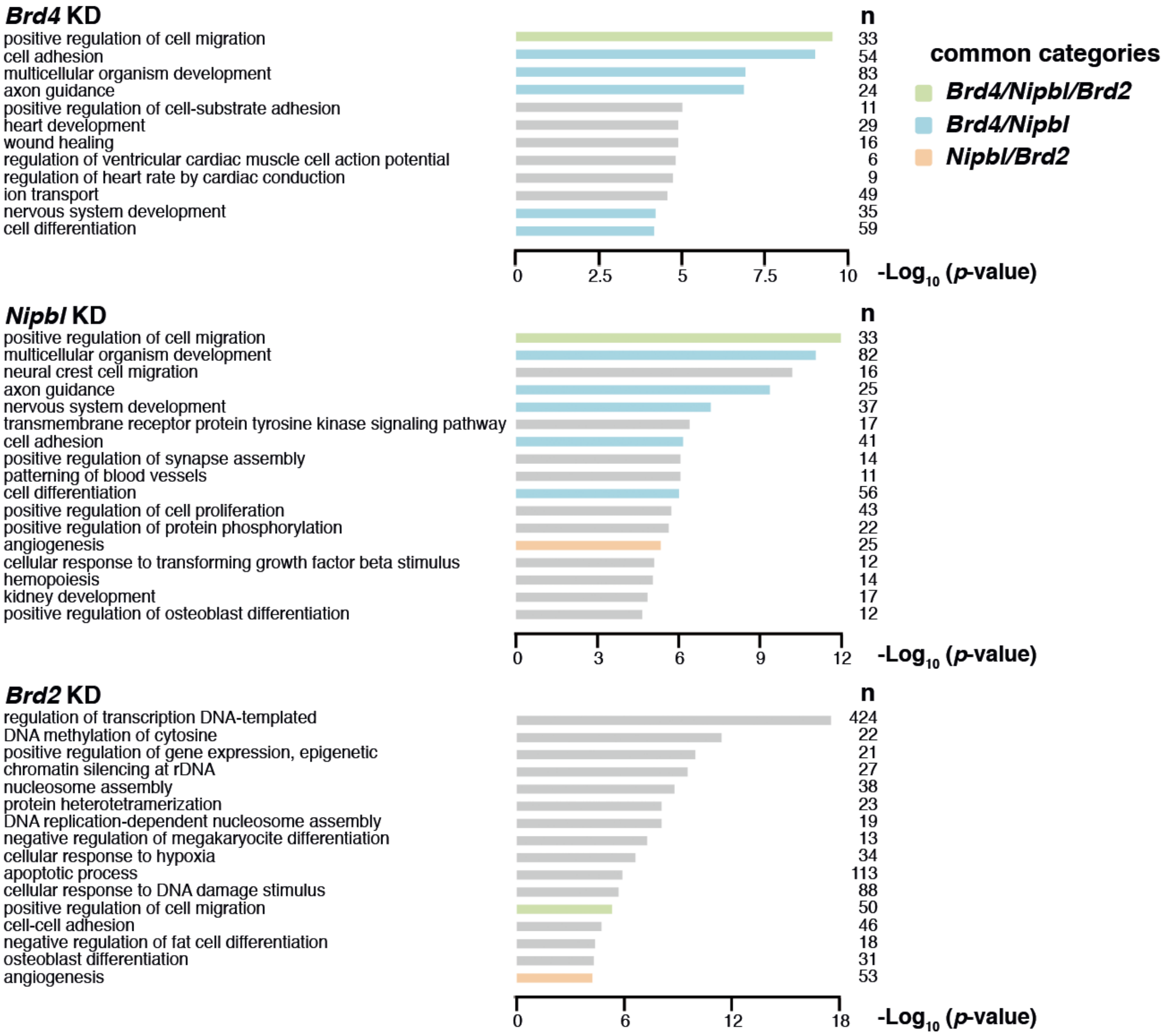

Supplementary Figure S3 NIPBL and BRD4 regulate a common set of genes involved in relevant developmental processes. Main GO categories comprising misregulated genes upon knocking down *Nipbl*, *Brd4* and *Brd2*. A *p*-value cut-off of 8.0 10^{-5} was established for the different knockdown analysis. Common categories are marked in colours as follows: green, common categories of misregulated genes upon depletion of the three proteins; blue, common categories of misregulated genes upon depletion of NIPBL and BRD4; orange, common categories of misregulated genes upon depletion of NIPBL and BRD2. n indicates the number of genes in the different categories.

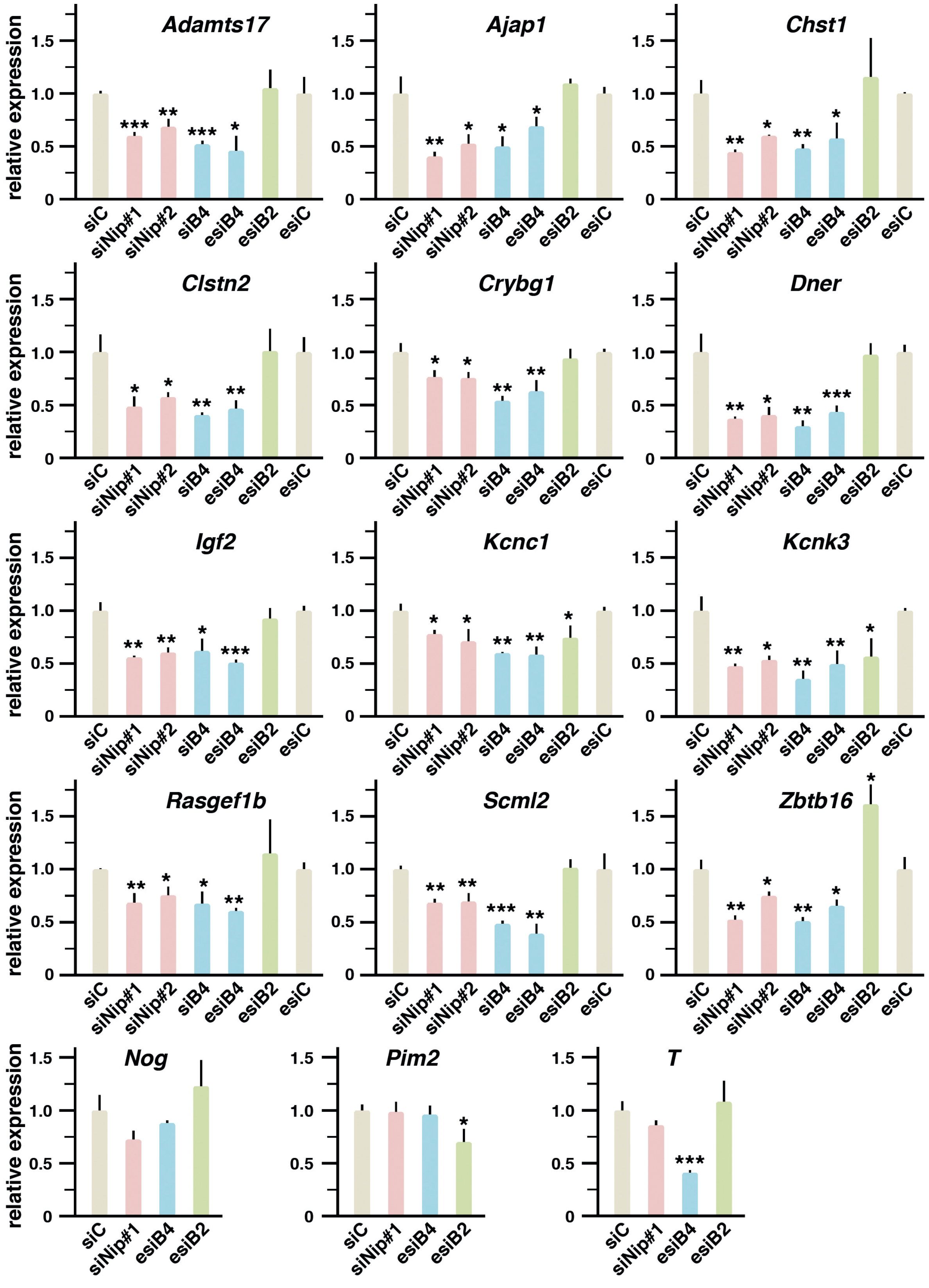
Adamts17
Ajap1
Chst1
Clstn2
Crybg1
Dner
Igf2
Kcnc1
Kcnk3
Rasgef1b
Scml2
Zbtb16
Nog
Pim2
T
relative expression
0
0.5
1.0
1.5
siC
siNip#1
siNip#2
siB4
esiB4
esiB2
esiC

Supplementary Figure S4 Expression analysis validates RNA-Seq data for a selection of genes. Twelve downregulated genes under NIPBL or BRD4 depletion conditions and 3 control genes were assessed for gene expression through qPCR after transfection of the indicated siRNA and esiRNA molecules. siC, Control siRNA; siNip#1, *Nipbl* siRNA #1; siNip#2, *Nipbl* siRNA #2; siB4, *Brd4* siRNA; esiB4, *Brd4* esiRNA; esiB2, *Brd2* esiRNA; esiC, Control esiRNA. Relative levels of expression are represented. Values are means ± s.d. of 3 independent experiments analyzed in triplicate. Statistical significance of each condition in comparison with the corresponding control was analyzed by Student's t-test (*$p < 0.05$, **$p < 0.01$, ***$p < 0.001$).
