# Supplementary material for: The Cornelia de Lange Syndrome-associated factor NIPBL interacts with BRD4 ET domain for transcription control of a common set of genes
Source: Cell Death Dis. 2019 Jul 18;10(8):548. doi: 10.1038/s41419-019-1792-x (PMC6639259; doi:10.1038/s41419-019-1792-x)
Supplement: Supplementary file 4 — Supplementary Table S3 [file 41419_2019_1792_MOESM4_ESM.docx]

**Supplementary Table S3** Primers used for RNA and ChIP analysis

| **RNA P19** | **forward (5’ > 3’)** | **reverse (5’ > 3’)** |
| --- | --- | --- |
| *Adamts17* | AGCGCTGCAAAGTAGTGACA | GGTCTCCACTGTGTGTTCGT |
| *Ajap1* | GCACAACGGAGCCTTCCA | GAGCGCAGCGATGACCAT |
| *Chst1* | GCTTCCGCCTCTCCTATGAC | GGCCGGATTCTTCAGTTCCT |
| *Clstn2* | TTGATGCCACCAACTCAACTG | ACCTGCTCATAGCGGTTCATG |
| *Crybg1* | AAGTCCGACGGCAGGATCTA | GACCATGGCTTCCCACACTT |
| *Dner* | CACCCAGGAGCCTGACATAA | GCATTCCCACAGGCAACATC |
| *Igf2* | GCTTGTTGACACGCTTCAGT | AAGCAGCACTCTTCCACGAT |
| *Kcnc1* | CATCTGGGCGCTGTTCGA | GTTGTGATGGAGACCAGGATGAA |
| *Kcnk3* | CTTCGCCATCACCGTCATCA | GCGCGTAGAACATGCAGAAC |
| *Rasgef1b* | TGGGTCTCTGGAAGCCCTTA | GAGCTCGTACGGATGCATGA |
| *Smcl2* | CCCGAGACATCTTCCCAGTT | GTTCTTCTTTTGCGCCTTGGT |
| *Zbtb16* | GACGCACTACAGGGTTCACA | GGGCAGTATTCCGTGCAGAT |
| *RpLp0* | CCAGGCTTTGGGCATCAC | CTCGCTGGCTCCCACCTT |
| **RNA Droso.** |  |  |
| *Shaw* | TTTCCAGCCCGAAAAGTTGG | TTGCTTGCGGAGAACCAAAC |
| *Task7* | GCTGAGCTTGGTCTTCATCC | TCTCTTGGCATCCTCTGCTT |
| *SoxN* | CCATACCGTTCTGCTTCGAT | CCCGTAATTGGCAACAGTTT |
| *bsh* | AGAAACGCGGTGAAAAGTGC | TGTTGCGACGGATGTTTGTG |
| *wg* | TGTTGTGTCCCATGATTGCC | TGCGTATGCCGATATTGCTG |
| *arm* | TCTGCTGCAACGAAACAACG | CTGCATCCGAAAGATTGCGG |
| *HDAC3* | TATGGACTTCTACCGGCCCA | CCTTGGTGCTCAGGGAGAAG |
| *RpL32* | ACAGGCCCAAGATCGTGAAG | CTTGCGCTTCTTGGAGGAGA |
| **ChIP** |  |  |
| *Ajap1* | ACTCAAGCCCAGTGCGGTTA | CTCGGTAGGTGGGCAAAGTTT |
| *Kcnc1* | GCAGCTGACCTTCCCCTAAA | CGGTATCATCTCAGCGGGAAA |
| *Kcnk3* | TCGCCGGCTCCTTCTACTT | GGCGAGTGTAGTTACCGATGGT |
| *Rasgef1b* | GCAAGTGCTTCGTGGTTCCT | GTAAATACCGGCTCGACTCGTT |
| *AJAP1* | CCAGGGCCAGATGGAAGAG | AAGTTTGCCGAGAAGGGTCTTT |
| *KCNC1* | CCACAGCCACTTCGACTATGAC | CTTGCCCGTGCGGTAGTAG |
| *KCNK3* | CGCCGGCTCCTTCTACTTC | CGAGCCGTTACCGATGGT |
